# Linguistic Validation and Cross-Cultural Adaptation of the Shoulder Telehealth Assessment Tool for Filipino Patients with Musculoskeletal Shoulder Condition: Cross-Sectional Study

**DOI:** 10.2196/67974

**Published:** 2026-01-20

**Authors:** Jeffrey Arboleda, Sharon Ignacio, Jose Alvin Mojica, Carl Froilan Leochico

**Affiliations:** 1 Department of Rehabilitation Medicine, Philippine General Hospital, University of the Philippines Manila, Taft Avenue, Ermita, Metro Manila, 1000, Philippines, 63 9282480184; 2 Department of Physical Medicine and Rehabilitation, St. Luke's Medical Center, Metro Manila, Philippines; 3 Department of Medicine, Division of Physical Medicine and Rehabilitation, Toronto Rehabilitation Institute, University Health Network, Toronto, ON, Canada

**Keywords:** cross-cultural adaptation, linguistic validation, musculoskeletal rehabilitation, shoulder examination, Shoulder Telehealth Assessment Tool, telerehabilitation, STAT

## Abstract

**Background:**

Telerehabilitation has been widely adopted to meet the growing rehabilitation demand, but it is often limited by unstable internet connection, poor audiovisual resolution, and difficult virtual assessment. The Shoulder Telehealth Assessment Tool (STAT), a comprehensive, patient-led, preconsultation shoulder physical examination pictorial guide, was developed to address these limitations by easing the communication of instruction during the consultation and potentially removing the need for video calls.

**Objective:**

This study aimed to develop a linguistically valid and culturally appropriate Filipino version of STAT and to evaluate its content validity, internal consistency, understandability, and ease of use.

**Methods:**

A cross-sectional study on the Filipino STAT was conducted in three phases: (1) linguistic validation by experts, (2) cross-cultural adaptation through pretesting of 12 participants diagnosed with a musculoskeletal shoulder condition at the Philippine General Hospital, and (3) pilot study on 47 participants of the same population.

**Results:**

The Filipino STAT had an excellent content validity (scale validity index=0.80‐0.97), excellent interrater reliability (κ coefficient=0.82‐1.00), and good internal consistency (Cronbach α=0.87). Understandability was found to be excellent for pain and activity (98%), good for range of motion and special tests (85%), and poor for strength (37%). However, 24% (11/46) of participants perceived the tool difficult to understand with the use of some Tagalog words as the primary barrier, followed by non-familiarity with the tool and difficulty reading the text.

**Conclusions:**

Development of the Filipino STAT through a rigorous linguistic validation and cultural adaptation has produced a culturally appropriate, valid, and reliable tool. Pain and activity, range of motions, and special test subdomains are suitable for clinical assessment, while strength subdomain needs further improvement in understandability.

## Introduction

Telemedicine is the use of telecommunication technologies to deliver health care, public health, and health education services remotely [1]. Telerehabilitation is a branch of telemedicine specifically aimed at delivering rehabilitation services. Being an archipelago with limited health care professionals and facilities, the Philippines uses these innovative health care delivery services to improve health access for Filipinos. Telerehabilitation in the Philippines was first adopted in the context of community-based rehabilitation in 2017 [2] and was widely used in 2020 during the COVID-19 pandemic [3]. With the improving COVID-19 situation, telerehabilitation remains a viable solution to delivering rehabilitation services in far-flung areas of the country [4].

However, telerehabilitation is not without limitations, such as unstable internet connection, lack of confidence in establishing clinical diagnosis virtually, limited time allotment per patient, and poor audiovisual resolution [5]. Specifically, in the administration of virtual physical examination, rehabilitation professionals face difficulties in the conduct of special tests, range of motion (ROM) assessment, and strength testing, among others [3]. These challenges have opened opportunities for innovation in telerehabilitation.

One of these innovations was the development of the Shoulder Telehealth Assessment Tool (STAT), which is a comprehensive patient-led shoulder physical examination pictorial guide done prior to the actual teleconsultation, aimed to improve clinical efficiency [6]. It is the first published patient-reported outcome measure to simulate the performance of in-person physical examination, which includes special tests for screening of different shoulder pathologies [7]. A validated visual analog scale (VAS) [8], single assessment numeric evaluation (SANE) [9], and motion analysis and range of motion studies on activities of daily living [10] have also been integrated into the tool. The first subcategory of the STAT is composed of 3 questions for pain and activity. Both VAS and SANE are patient-reported. The VAS ranges from 0=no pain to 10=maximum pain, while the SANE is scored from 0% to 100% of normal. Meanwhile, the current level of daily activity is a nominal score (ie, unaffected sleep, full work, and full recreation or sport). The ROM testing has 9 questions that are answered with either yes (movement completed) or no (movement not completed). There are 5 items for the shoulder special tests answerable by yes or no, and finally 3 items for strength that are answered with either painful, weak, both, or none. Overall, there are a total of 20 questions in the STAT that can be completed in 30 to 45 minutes. In the Philippines and numerous other countries where Filipinos are found, the STAT can potentially improve the accuracy of virtual physical examination techniques for the shoulder, provide better rehabilitation care for patients who do not have stable internet connections or video call capacity, and optimize the delivery of telerehabilitation services. This may also be used by providers from various health care disciplines, such as physiatry, physical therapy, occupational therapy, orthopedics, rheumatology, and pain management, among others, which routinely conduct musculoskeletal examination of the shoulder.

In the Philippines, shoulder conditions have been found to be prevalent among Filipino office workers [11], as well as migrant workers [12]. Currently, the STAT is available in English [6] and has not been translated into any other language. Language and cultural differences call for a careful adaptation of health outcome measures to accurately reflect the cultural nuances and context of the target language version [13]. Hence, this study aimed to develop a linguistically valid and culturally appropriate Filipino version of the STAT and determine its content validity, internal consistency, understandability, and ease of use.

## Methods

### Study Design

This cross-sectional study used a mixed methods research design on the linguistic validation and cross-cultural adaptation of the Filipino STAT.

### Participants

The target population was Filipino adults (aged at least 18 years) with unilateral shoulder pain for at least 6 weeks who consulted in-person or through telemedicine at the Philippine General Hospital (PGH)—Outpatient Department of Rehabilitation Medicine from January 2023 to June 2023. Based on the Department census during that period, there were 304 patients diagnosed with shoulder pathology, such as adhesive capsulitis, rotator cuff injury, and myofascial pain syndrome. The participants should have access to a stable internet and a device with video call capacity and be able to understand written instructions in Filipino or Tagalog, which is the Philippines’ most commonly used language. Participants with a history of trauma, suspicion or diagnosis of upper extremity fracture, severe cognitive impairment, known psychiatric comorbidity, cerebrovascular disease, cervical radiculopathy, brachial plexus injury, upper extremity peripheral nerve injury, complex regional pain syndrome, and prior shoulder, neck, and breast surgery were excluded. The participants were allowed to withdraw from the study for any reason.

### Sampling

The sample size for the pretest was based on a generally recommended sample size of 12 from the target population based on feasibility and precision of estimates for succeeding studies [14]. The sample size for a pilot study to determine internal consistency through Cronbach α was 46 [15]. Systematic randomized sampling was done where every seventh patient was selected.

### Study Procedure

#### Overview

**Figure 1.**
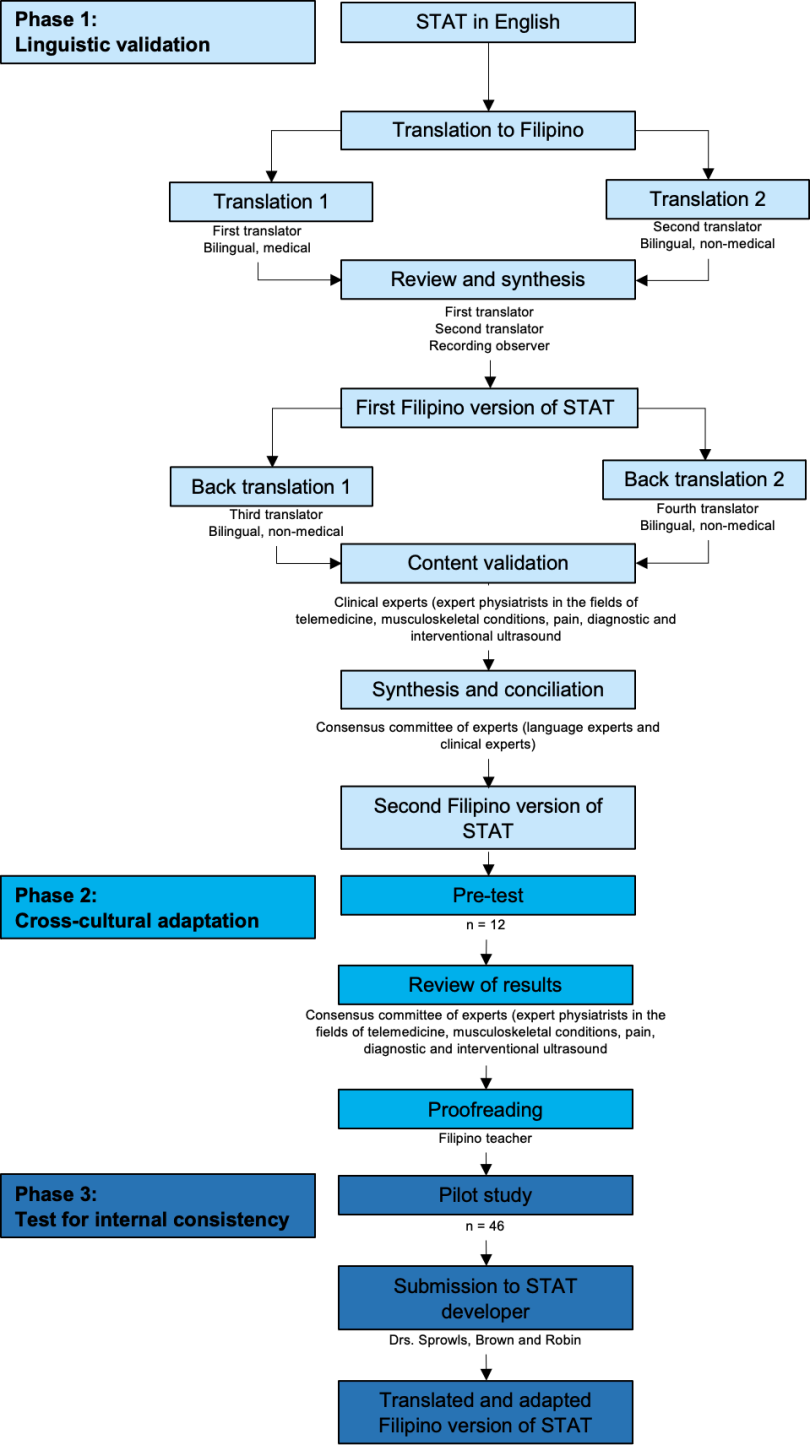
This is a flowchart adapted from Lorca et al [17] for linguistic validation and cross-cultural adaptation [6]*.* STAT: Shoulder Telehealth Assessment Tool.

Adapting the recommendations on linguistic validation and cross-cultural adaptation of self-reported measures, the study procedure was divided into two phases [16,17]: (1) translation of the STAT from the original English to the Filipino version; and (2) cross-cultural adaptation through pretesting. An additional phase was added for internal consistency testing of the final version of the Filipino STAT (Figure 1). Meetings, where necessary, were all done virtually, and permission to record each online meeting was sought from all participants.

#### Phase 1: Linguistic Validation

##### Step 1: Permission From the Instrument’s Authors

Permission for linguistic validation and cross-cultural adaptation into the Filipino language was obtained from the STAT developers prior to initiation of translation.

##### Step 2: Forward Translation

The forward translation was independently done by 2 bilingual translators. The first forward translator (T1) was a licensed physical therapist with a Master’s degree in physical therapy who provided a “reliable equivalence from a clinical perspective,” while the second forward translator (T2) was a university instructor and a representative from the *Sentro ng Wikang Filipino* (National Center of the Filipino Language) with no medical background who served to “reflect the language used by the population.”

##### Step 3: Review and Synthesis

The translators, together with a recording observer, convened to synthesize their versions and arrive at a consensus version. Translation and synthesis forms were used throughout the linguistic validation process by the translators and recording observer to document the translated version, rationale for changes, and any disputes or challenges in doing the translation or synthesis.

##### Step 4: Backward Translation

Backward translation was independently done by another pair of bilingual translators (B1 and B2), who happened to be high school Filipino language teachers and were not familiar with the original version. This step was done to prevent information bias and to detect any errors in the forward translation.

##### Step 5: Content Validation

The first forward- and backward-translated version of the Filipino STAT was then assessed for content validity independently by clinical experts using a content validation form. The experts were composed of 6 board-certified local physiatrists with expertise in the fields of sports medicine, musculoskeletal ultrasound, interventional physiatry, telerehabilitation, clinical anatomy, and kinesiology. The form was composed of a yes or no scale for clarity, 4-point Likert scale for relevance, and a comment section for each item.

##### Step 6: Synthesis and Conciliation

All versions of the Filipino STAT available so far and the completed content validation forms (Multimedia Appendix 1) were then reviewed by a consensus committee of experts to arrive at a prefinal version. The consensus committee consisted of all 4 translators (T1, T2, B1, and B2) from the previous steps and the 6 clinical experts who participated in the content validation step. They all met virtually and collectively decided and agreed on each item through the Expert Consensus Committee Guide on Equivalence Form (Multimedia Appendix 2), wherein semantic, idiomatic, experiential, and conceptual equivalence were assessed between the forward- and backward-translated English and Filipino versions. Semantic equivalence pertains to the use of words that have similar, unique meaning in both cultures. Idiomatic equivalence pertains to the use of equivalent colloquialisms and idioms in both cultures. Conceptual equivalence pertains to the use of words or phrases with equivalent conceptual meanings in both cultures, and experiential equivalence pertains to experiences elicited that are consistent or equivalent in both cultures. The form contained a yes or no scale on each type of equivalence, a column for rewording suggestions, and another column for other comments and suggestions to meet cultural equivalence.

### Phase 2: Cross-Cultural Adaptation

#### Step 1: Pretesting

A pretest on 12 adult Filipino patients with shoulder pain was done using the prefinal version of the translated tool through video consultation to simulate the actual STAT procedure and to avoid unnecessary risk of contagion during the pandemic (time of study).

A think-aloud protocol during each video call with the principal investigator or research assistant using an encrypted platform, such as Zoom (Zoom Communications, Inc) or Viber (whichever the participant preferred), was used. The think-aloud protocol entailed each participant to read, perform, and answer the Filipino STAT while verbalizing their thoughts. This was done twice for each question: first without the pictorial guide; and second with the pictorial guide. The trial without the pictorial guide was done to assess the feasibility of the Filipino STAT Text Version for patients with no access to smartphones. An observational checklist (Multimedia Appendix 3) was filled out by the principal investigator to document the participant response, understandability of the tool, and ease and accuracy in performing the Filipino STAT, noting participants’ quality of movements and compensatory movements. The principal investigator also asked each participant open-ended interview questions (Multimedia Appendix 4) on their experience (including perceived barriers and facilitators) in using the tool and their suggestions in improving it.

#### Step 2: Review of Results

General and question-specific errors and suggestions from the pre-test participants were reviewed by the committee in developing the final version of the Filipino STAT.

#### Step 3: Proofreading

The resulting version was then proofread by a Filipino language teacher to correct any spelling and grammatical errors. All translation forms and STAT versions were sent to the original authors for their review and feedback.

### Phase 3: Internal Consistency Testing

Finally, all 46 participants underwent the same process as pretesting using the final version of the Filipino STAT for the pilot study to determine the internal consistency of the tool.

### Data Analysis

Content validation was determined through computation of item-level and scale-level content validity indices based on average and universal agreement methods. Sociodemographic data were reported using descriptive statistics. The internal consistency of participant responses was determined through Cronbach α. This was evaluated separately for items in VAS, SANE, ROM, strength tests, and special tests using STATA 16.1/IC (StataCorp LLC). Considering ≥90% as excellent, 89% to 70% as good, 69% to 50% as fair, and ≤49% as poor, understandability was assessed using the worst-performing item in the observational checklist.

Interview responses were uploaded to NVivo 12 (Lumivero) for organization and thematic analysis of unstructured data pertaining to understandability. An inductive approach was employed where the data content directed the development of themes. The members of the research team were all physiatrists and had broad experience in musculoskeletal evaluation and telerehabilitation in clinical practice and research. JA was a rehabilitation medicine resident trained during the height of telerehabilitation during the COVID-19 pandemic. CFL spearheaded several research endeavors on telerehabilitation in the Philippines. SI and JM were the chairs of the Department of Rehabilitation Medicine with extensive teaching, research, and clinical experiences.

### Ethical Considerations

The study was approved by the University of the Philippines Manila Research Ethics Board (UPMREB Code 2022-0516-01). Informed consent was secured through Google Forms, written and explained in Filipino either by the principal investigator or research assistant. Study introduction, objectives, procedure, duration, risks, benefits, incentives, and contact information were discussed with the participants. Participants were also assured of the confidentiality of their information, the voluntary nature of participation, and their right to refuse at any point of the study. The translators, recording observers, and Filipino teacher were appropriately compensated with Philippine ₱1000 each (US $20). All phase 2 participants were compensated with Philippine ₱200 (US $4) worth of prepaid load as reimbursement for internet fee for the video call interview and answering of online forms. The clinical experts did not receive remuneration and were acknowledged in this paper.

## Results

### Forward Translation

Both forward translators had challenges in translating the strength and special tests questions due to absence of direct translation of some English words to Filipino. Hence, both translators resorted to rephrasing some English sentences in Filipino. During synthesis, T1 shared the technical context of the subtests, while T2 suggested the use of understandable albeit literal translations of some words. For item Q9 (ROM) (Table 1), the use of the phrase “*itupi ang siko*” was selected over “*itupi ang braso*” based on T1’s input to pertain to the correct anatomical joint that does flexion. On the other hand, for item Q12 (special test), “*idiin ang kamay sa tiyan*” was selected over “*pindutin ang tiyan*” based on T2’s input on the context of pressing the belly. Items that resulted in differences between T1 and T2, as well as the final terminology agreed upon during synthesis, are presented in Table 1.

**Table 1.** Results of the forward translation and synthesis steps.

Item	T1^a^	T2^b^	Prefinal STAT^c^
Pain and activity			
Q1	*Lebel* *Pinakamasakit na naramdaman sa iyong buhay*	*Antas* *Pinakamasakit*	*Lebel* *Pinakamasakit*
Q3	*Dalas ng pisikal na aktibidad* *Hindi apektado ang pagtulog* *Nagtatrabaho nang buong araw* *Nagagawa ang panlibangang aktibidad o isports*	*Antas ng pang-araw-araw na aktibidad* *Di naaabalang pagtulog* *Panay trabaho* *Panay lingan/ isports*	*Antas ng pang-araw-araw na aktibidad* *‘Di naaabalang pagtulog* *Nakakapagtrabaho nang buong araw* *Nagagawa ang panlibangang aktibidad o isports*
Range of motion	*Saklaw ng galaw*	*Saklaw ng paggalaw*	*Saklaw ng paggalaw*
Instructions	*Kilos*	*Paggalaw*	*Paggalaw*
Q4	*Taas ng ulo*	*Tuktok ng ulo*	*Taas ng ulo*
Q7	*Bulsa ng pantalon sa parehong panig*	*Bulsa sa likod*	*Bulsa sa likod ng apektadong balikat*
Q8	*Ibabang likod*	*Ibabang bahagi ng iyong likuran*	*Ibabang bahagi ng iyong likuran*
Q9	*Braso ay nakaharap sa pader* *Itupi ang siko* *Ilapat*	*Nakatagilid sa pader* *Itupi ang braso* *Idikit*	*Nakatagilid sa pader* *Itupi ang braso* *Ilapat*
Strength			
Instructions	*Bigyan ng pwersa* *Mahina ba ang pakiramdam?* *Wala sa mga nabanggit*	*Habang nilalabanan ang bigat* *Nanghihina ba?* *Wala*	*Bigyan ng pwersa* *Mahina ba ang pakiramdam?* *Wala*
Q10	*Gamit ang kamao* *Idiin ang palad ng kamay sa masakit na braso*	*Nakakuyom ang kamao* *Idiin ang mga ito sa isa’t isa*	*Gamit ang kamao* *Idiin ang palad ng kamay sa masakit na braso*
Q11	*Hilahin* *Direksyon ng kabilang braso*	*Kapitan* *Gumalaw pakanan at pakaliwa*	*Hilahin* *Direksyon ng normal na braso*
Q12	*Labanan* *Ilayo*	*Kapitan* *Palayo*	*Kapitan* *Ilayo*
Special tests			
Instructions	*Nakasaad na kilos at sasabihin mo*	*Maniobra na makakapagsabi sa amin*	*Mga kilos na makakapagsabi sa amin*
Q13	*Iangat ang buong braso*	*Itaas ang kamay*	*Itaas ang kamay*
Q14	*Umabot lagpas sa kabilang balikat*	*Ilagay ang inyong apektadong braso sa harap ng inyong dibdib*	*Umabot lagpas sa kabilang balikat*
Q15	*Pindutin*	*Idiin ang kamay*	*Idiin ang kamay*
Q16	*Ilapat*	*Idikit*	*Ilapat*
Q17	*Itaas ang braso na parang may hawak na plato* *Dahan-dahang itulak pababa*	*Iangat ang braso sa harap ng inyong dibdib at ilahad ang palad* *Bahagyang diinan*	*Itaas ang braso na parang may hawak na plato* *Dahan-dahang itulak pababa*

a T1: forward translator 1.

b T2: forward translator 2.

c STAT: Shoulder Telehealth Assessment Tool.

### Backward Translation

Similar to the forward translators, the back translators did not apply word-for-word translation in strength and special test questions to simplify the items and make them easier to understand. The back translation versions were generally consistent with the original English version, with no significant differences, as agreed upon by the consensus committee.

### Content Validation of Prefinal STAT

The instructions in all subdomains, except for strength, were clear to the experts. The items on pain and activity (Q1) and range of motion (Q4, Q5, and Q6) were clear to all experts. Content validity through the scale validity index was measured using the item-level content validity index (0.97) and using universal agreement (0.80). κ values across all items ranged from 0.82 to 1.00, indicating excellent inter-rater reliability.

### Cultural Equivalence

Pertinent findings from the discussion of the consensus experts on equivalence are summarized in Table 2.

**Table 2.** Pertinent results from the expert consensus discussion on equivalence^a^.

	SE^b^	IE^c^	CE^d^	EE^e^	Comments
Pain and activity					
Q1	✓	✓	✓	✓	Inconsistent: not a question
Q2	✓	✓	✓	✓	Inconsistent: not a question
Q3	✓	✓	✓	X	Inconsistent: not a question Vague: choices not in one spectrum EE: did not include informal work
Range of motion					
Q7	✓	✓	X	✓	CE: not relatable to everyone
Special test					
Instruction	✓	X	✓	✓	IE: Test demands active patient response
Q13	✓	✓	✓	✓	Safety: for severely painful shoulder Unclear: confusing literal translation
Q17	✓	✓	X	✓	CE: incomplete instruction for the test

a Checks (✓) indicate equivalence, while cross marks (X) indicate non-equivalence.

b SE: semantic equivalence.

c IE: idiomatic equivalence.

d CE: conceptual equivalence.

e EE: experiential equivalence.

To facilitate consistency of the translations throughout the tool, items Q1 to Q3 (pain and activity) were converted from declarative statements into questions. Choices for item Q3 (sleep, work, and recreation or sports) were deemed to pertain to different aspects of activities and were thus converted to stand-alone questions for each activity (Q3A, Q3B, and Q3C). To facilitate clarity, the literal translation of item Q13 was rephrased for easier understanding. To ensure patient safety when using the tool, precautions for severely painful shoulders were added to the instructions for the special test subdomain.

To facilitate experiential equivalence, item Q3B’s use of the term “*nakakapagtrabaho*” (able to work) was supplemented by the modifier “*gawaing bahay*” (house chores) to be more encompassing of the different types of work in the Filipino culture. To facilitate conceptual equivalence, item Q7’s (ROM) use of “*bulsa sa likod ng apektadong balikat*” (back pocket on the affected side) was changed to “*pigi*” (buttock) as it pertains to the same area and is more relatable and easily understandable. Likewise, item Q17 was clarified with the phrase “*paharap sa lebel ng braso*” (forward raising to arm level) to demonstrate shoulder flexion to 90 degrees as in the Speed test. To facilitate idiomatic equivalence, instruction for the special test subdomain was improved from “*na makakapagsabi sa amin*” (that may tell us) to “*upang malaman namin*” (so that we will know) as the test demands active patient response. Finally, the expert committee all suggested taking new pictures for the tool with a Filipino-looking model for the pictorial guide for it to be more relatable and also to facilitate minor improvements in the angle of the shots and designation of the task of each arm.

### Pretest

There were 12 participants in the pretest, with the majority being females (10/12, 83%), aged 50‐59 years (5/12, 42%) with a mean age of 53 (SD 12) years, married or cohabiting (5/12, 42%), and having finished tertiary education (8/12, 67%; Table 3). Most were unemployed (6/12, 50%), while those employed were engaged in nonhealth-related work (8/12, 67%). The monthly family income of participants was positively skewed, with the majority (7/12, 58%) earning Philippine ₱5000 to ₱10,000 (US $100‐$200).

**Table 3.** Sociodemographic profile of the participants.

Characteristics	Pretest (n=12)	Pilot (n=47)
Age group (y), n (%)		
19‐29	0 (0)	2 (4)
30‐39	2 (2)	5 (11)
40‐49	2 (2)	3 (6)
50‐59	5 (42)	21 (45)
≥60	3 (25)	16 (34)
Age (y), mean (SD)	53 (12)	55 (14)
Sex, n (%)		
Female	10 (83)	35 (75)
Male	2 (17)	12 (26)
Civil status, n (%)		
Single	4 (33)	10 (21)
Married or cohabiting	5 (42)	29 (62)
Separated or divorced	1 (8)	2 (4)
Widowed	2 (17)	6 (13)
Educational status, n (%)		
Primary	0 (0)	4 (9)
Secondary	4 (33)	15 (32)
Tertiary	8 (67)	26 (55)
Postgraduate	0 (0)	2 (4)
Employment, n (%)		
Student	0 (0)	2 (4)
Employed	4 (33)	15 (32)
Unemployed	6 (50)	23 (49)
Retired	2 (17)	7 (15)
Type of work, n (%)		
Health-related work	4 (33)	7 (15)
Nonhealth-related work	8 (67)	40 (85)
Family monthly income (Philippine ₱ [US $]), n (%)		
<5000 (<100)	2 (17)	21 (45)
5000-10,000 (100‐200)	7 (58)	9 (19)
10,001-20,000 (201‐400)	2 (18)	8 (17)
20,001-50,000 (401‐1000)	1 (8)	6 (13)
>50,000 (>1000)	0 (0)	3 (6)

Items on pain and activity (Q1 and Q3A-Q3C), ROM (Q2-Q6), and special tests (Q15) were performed correctly by at least 75% (9/12) of the participants independently. Up to 25% (3/12) of the participants needed cueing in the whole pain and activity subdomain and special tests (Q14). Less than 25% of the participants were able to perform ROM items Q8 and Q9, all strength items, and half of the items in the special test subdomain.

Six out of 12 (50%) participants reported an increase in pain upon performance of certain maneuvers (eg, items Q4, Q5, Q7, Q9, and Q14), although all were still able to complete the tasks. They were advised to seek outpatient consultation at the Rehabilitation Medicine Outpatient Clinic if the pain persisted.

Six (50%) participants found the tool easy to follow, while 5 (42%) found it difficult. Visual aids in the form of pictures and arrows were most helpful in making the tool easily understandable. The presence of a caregiver was helpful for two of the participants.

The use of some Tagalog words (such as *pigi* or buttocks in English) was the primary barrier for understanding the tool (Table 4). Five (42%) participants were content with the prefinal version of the tool and had no suggested changes. One (8%) participant suggested that the presence of a physician or a caregiver (at the least) was still necessary to guide patients and ensure accuracy and safety in following the tool. Finally, 6 (50%) participants reiterated the need for pictures, and 1 (8%) participant suggested improvement in the portrayal of movement in the pictures.

**Table 4.** Results from the thematic analysis related to understandability of the Filipino Shoulder Telehealth Assessment Tool (STAT) and factors that make it easy or difficult to understand.

	Pretest (n=12), n (%)	Pilot (n=46), n (%)
Ease of understanding		
Tool is easy	6 (50)	13 (28)
Use of pictures and arrows	10 (83)	11 (24)
Presence of caregiver	2 (17)	3 (6)
Tool is difficult	5 (42)	11 (24)
Use of some Tagalog words	3 (25)	7 (15)
Nonfamiliarity with the tool	1 (8)	3 (6)
Difficulty reading the text	2 (17)	3 (6)
Poor internet connection	2 (17)	2 (4)
Poor audiovisual setup	1 (8)	1 (2)
Not adept with technology	1 (8)	1 (2)
Absence of caregiver	0 (0)	1 (2)
Nonideal venue	1 (8)	1 (2)
Use of arrows	1 (8)	0 (0)

### Lessons From the Pretest and Development of the Final Filipino STAT

Difficulty reading the text of the tool and tendency to skip a specific question (Q3A) in a predominantly older adult population suggested inappropriate user interface design to the study team. While readers with advancing age have varying levels of possible age-related cognitive and visual decline, inclusivity was ensured in redesigning the tool [18]. These lessons were applied to the final version of the Filipino STAT (Multimedia Appendix 5) as follows: breaking down chunks of texts into bullet points, using Sans Serif typefaces with at least a 16-point font size, and providing white spaces and appropriate contrasts [18,19].

The conduct of special tests revealed an average of 73% (SD 14%) positive test findings (painful) that might have contributed to the increase in shoulder pain among pretest participants. This was addressed in the final version by placing the special test subdomain last as in clinical examination so any pain would not get in the way of the conduct of the other parts of the assessment.

Specific pretest errors could be classified as (1) misinterpretations observed in the subdomains of pain and activity (items Q2 and Q3C) and ROM (Q8); (2) differences in the semantic understanding of specific body parts, evident in ROM items Q1, Q4, Q5, Q7, and Q8; (3) difficulty understanding long instructions, which were evident in ROM item Q9 and all strength and special test items; and (4) inconsistent experiential equivalence in special test item Q17. Text qualifiers were added to misinterpreted items such as Q2, labeling 0=“normal” and 100=“hindi normal” (not normal), to avoid reversal of the scale. Items with different potential semantic meanings were significantly improved when pictorial guides were provided. Long, unclear instructions were broken down into short, step-by-step instructions with pictorial guides in each step. Finally, in item Q17 (Speed test), instead of holding a plate forward, most participants held a plate on the side, as a waiter would. The phrase was therefore changed from “*parang may hawak na plato*” (like holding a plate) to “*parang nanghihingi habang nakaunat ang siko*” (like reaching out or asking for something with an outstretched arm) to improve participants’ accuracy in performing the task and the relatability of the task in Filipino culture.

### Final Filipino STAT Pilot Study

There were 47 participants in the pilot study; one of them did not complete the video consultation and withdrew due to technical difficulties (ie, unstable internet) on the patient’s end. The majority of the participants were females (35/47, 75%), aged 50‐59 years (21/47, 45%) with a mean age of 55 (SD 14) years, married or cohabiting (29/47, 62%), and finished tertiary education (26/47, 55%; Table 3). Most were unemployed (23/47, 49%), while those employed were mostly engaged in nonhealth-related work (40/47, 85%). The monthly family income of the participants was positively skewed, with the majority (21/47, 45%) earning less than Philippine ₱5000 (US $<100).

For each of the items under the pain and activity subdomains, 35 (75%) to 43 (91%) participants were able to perform the tasks correctly. Meanwhile, some needed cueing from their caregiver to perform the tasks correctly, particularly for item Q2, where 11 (23%) participants needed help. With items Q3A and Q3C, 1 out of the 47 (2%) participants was not able to perform the task completely.

The ROM, strength, and special test subdomains were tested both without and with pictorial guides. For ROM-related items, a lot of participants were not able to perform the tasks correctly without a picture (Q2: chin, Q7: back pocket, Q8: lower back, and Q9: wall touching). In contrast, for those items with picture guides, there was a greater number of patients who were able to perform the tasks correctly, with little to no need of cueing from their caregivers. Four (8%) participants showed compensatory movements with some items, yet they answered yes when asked if they could do the tasks. Hence, they were considered incorrect task performances, with item Q8 being the most common item that resulted in compensatory movements.

Without pictorial guides, more than half of the participants were not able to perform all the tasks under the strength subdomain. Slightly more participants correctly performed the tasks for items Q11 and Q12 when shown pictorial guides. Moreover, the same scenario was observed for all items in the special test subdomain—having pictorial guides improved the number of participants able to perform the tasks correctly from 34 (74%) to 39 (87%) participants, while 2 (4%) to 6 (13%) participants needed cueing from their caregivers to be able to perform the tasks correctly.

Among the 46 participants who shared their experience with the final Filipino STAT, 13 (28%) found the tool easy to understand, while 11 (24%) found it difficult. The pictures and arrows were found to be most helpful for participants. Participant 47 remarked, “*maayos po binigay ang panuto at dahil sa larawan ay naintindihan po nang mabuti*” (the instructions were given properly, and because of the pictures, they were better understood). The use of some Tagalog words, however, was considered the primary barrier by the majority of the participants, followed by their nonfamiliarity with the tool and difficulty reading the text (Table 4). The same participant also remarked, “*May kahinaan po ako sa Pilipino, tulad po ng pigi po, hindi ko siya naintindihan*” (I have some difficulty in understanding Tagalog, for example, I did not understand the Tagalog word for buttock”).

### Content Validity and Internal Consistency Testing of the Final Filipino STAT

Content validity through scale validity indices was deemed excellent at 0.97 using the item-level content validity index and 0.80 using the universal agreement. The κ coefficients were excellent across all items, supporting that the degree of agreement among the experts was beyond chance [20]. A Cronbach α score of 0.87 indicated that all test items were unidimensional, or that performance on the items could be explained in terms of a single underlying factor (Multimedia Appendix 6).

## Discussion

### Principal Findings

The Filipino STAT has an excellent content validity (scale validity index=0.80‐0.97), excellent interrater reliability (κ coefficient=0.82‐1.00), and good internal consistency (Cronbach α=0.87). Its understandability is excellent for pain and activity (98%), good for ROM and special tests (85%), and poor for strength (37%).

### Comparison With Prior Work

The findings of this study are consistent with the existing literature on shoulder teleconsultation psychometric properties. Internet assessment of rehabilitation outcomes such as pain, ROM, muscle strength, and functional assessment had good concurrent validity. There is a strong agreement between virtual and in-person examination for ROM (87.4% agreement; *x*^2^=30.782; *P*<.001), and diagnosis (85.1% agreement; κ=0.82, 95% CI) [21-23]. Further studies on the Filipino STAT compared against in-person examination or imaging modalities may be done to ascertain concurrent validity of the tool.

### Strengths and Limitations

Since a direct translation of a tool from an original English version may not reflect the cultural nuances and context of another culture [13], this study observed a rigorous and standard linguistic validation and cross-cultural adaptation to ensure a culturally appropriate, valid, and reliable translation. Although the original STAT has not been validated as a whole tool, the Filipino-translated tool has been subjected to content validity and internal consistency testing in this study. The collective inputs from the consensus committee of experts, the understandability questionnaire, and qualitative data from the participants ensured that the final tool can be used in the clinical setting.

There are some limitations to this study. First, the assessment of ease of understanding of the tool using the open-ended questionnaire may have been confounded by the consecutive performance of the tool without and with a pictorial guide, as well as the videoconsultation study setup. The test-taking that was twice as long, with no visual aid provided at the start, could have made the consultation more difficult for the participants. The barriers to the videoconsultation study setup were consistent with previous literature, such as unstable internet connection and poor audiovisual resolution [5]. In the envisioned clinical performance of the Filipino STAT, only the pictorial guide will be performed before the actual consultation, thereby eliminating these concerns.

Second, understandability per subdomain was excellent for pain and activity, good for ROM and special tests, and poor for strength. The incidence of compensatory or trick movement was accounted for in this study and was most frequently seen in the ROM subdomain, particularly item Q8 (50 degrees of internal rotation).

Finally, such as in an in-person physical examination, incorrect performance of a virtual test, from poor understandability or compensatory movement, makes its results invalid and questionable. This shall serve as a reminder that the Filipino STAT is not meant to replace actual in-person consultations and should just aid the clinician in assessing the patient and make clinical work efficient. The clinician must be prudent in confirming the initial Filipino STAT findings during the actual consultation, as necessary. Further studies may consider the use of instructional videos to improve understandability of the tool, especially for the strength subdomain.

### Future Directions

Both pretest and pilot study findings reveal significant improvements in the correct performance of the Filipino STAT tasks with pictorial guides, compared to none. The majority of the participants from both phases of the study also shared a positive perception of the use of pictures and arrows and the presence of a caregiver. Thus, it is the study’s recommendation to use the Filipino STAT with a pictorial guide, as intended in the original tool [6]. The association of the presence of a caregiver in the successful performance of the Filipino STAT was beyond the scope of this present study. Nonetheless, having a caregiver around may help with the conduct of the Filipino STAT but should not discourage those who do not have an available caregiver.

### Conclusions

The development of the Filipino STAT through a rigorous linguistic validation and cultural adaptation ensured a culturally appropriate, valid, and reliable translation. Understandability and ease of understanding by end-users are also critical to assess in patient-reported outcome measures. The pain and activity, ROM, and special test subdomains of the Filipino STAT may be used for clinical assessment, while the strength subdomain needs further improvement on understandability.

## Supplementary material

10.2196/67974 Multimedia Appendix 1 The content validation form.

10.2196/67974 Multimedia Appendix 2 The expert consensus committee guide on equivalence.

10.2196/67974 Multimedia Appendix 3 The observational checklist.

10.2196/67974 Multimedia Appendix 4 The open-ended interview questions.

10.2196/67974 Multimedia Appendix 5 The final Filipino Shoulder Telehealth Assessment Tool (STAT).

10.2196/67974 Multimedia Appendix 6 Supplemental dataset to a full manuscript published in the *J Med Internet Res*.

## Abbreviations

ROM: range of motion
SANE: single assessment numeric evaluation
STAT: Shoulder Telehealth Assessment Tool
VAS: visual analog scale
