# Supplementary material for: Linguistic Validation and Cross-Cultural Adaptation of the Shoulder Telehealth Assessment Tool for Filipino Patients with Musculoskeletal Shoulder Condition: Cross-Sectional Study
Source: JMIR Rehabil Assist Technol. 2026 Jan 20;13:e67974. doi: 10.2196/67974 (PMC12818489; doi:10.2196/67974)
Supplement: Multimedia Appendix 1 [file rehab-v13-e67974-s001.pdf]

Content Validation Form for STAT

This questionnaire contains 20 items related to shoulder assessment.
We need your expert judgment on the **degree of relevance and clarity of each item to the measured domain**. Please base your review on the domain and definition that are provided to you. You may put your rewording suggestions and other comments/ suggestions on the succeeding columns.
Degree of relevance:
1 = the item is not relevant to the measured domain
2 = the item is somewhat relevant to the measured domain
3 = the item is quite relevant to the measured domain
4 = the item is highly relevant to the measured domain

Item from Filipino Version of STAT	Relevance				Clarity		Rewording Suggestion	Other Comments/ Suggestions
	4	3	2	1	Yes	No		
**<u>Domain 1: Pain</u>**<br>Definition: “an unpleasant sensory and emotional experience associated with actual or potential tissue damage or described in terms of such damage.”[1]								
**<u>PANANAKIT</u>**<br>1. Pakibilugan ang numerong katumbas ng karaniwang antas ng sakit na nararamdaman sa araw-araw:<br>0 1 2 3 4 5 6 7 8 9 10<br>**Walang sakit** **Pinakamasakit**								
**<u>Domain 2: Functional Limitation</u>**<br>Definition: “issues surrounding mobility, ADL, instrumental activities of daily living (I-ADL), communication, cognition, work, and recreation, among others”[1]								
**<u>MGA GAWAIN</u>**<br>2. Markahan kung gaano ka-normal ang pakiramdam ng iyong apektadong balikat ngayon. Bilugan ang naaangkop na porsyento (mula 0% hanggang 100%, kung saan 100% ang katumbas ng normal).<br>0 10 20 30 40 50 60 70 80 90 100								
3. Pakibilugan ang inyong kasalukuyang antas ng pang-araw-araw na aktibidad:<br>**‘DI NAABALANG PAGTULOG** **NAKAKAPAGTRABAHO NG BUONG ARAW** **NAGAGAWA ANG PANGLIBANGAN NA AKTIBIDAD O ISPORTS**								
**<u>Domain 3: Range of Motion</u>**<br>Definition: “function of joint morphology, capsule and ligament integrity, and muscle and tendon strength”[1]<br>Forward Flexion: Able to actively take hand to (chest, chin, nose, top of head, back of head) corresponding to (30, 35, 60, 105, 110 degrees of forward flexion)<br>Abduction: Able to actively take hand to (chest, chin, nose, top of head, back of head) corresponding to (0, 30, 55,110, 125 degrees of abduction)<br>Adduction: Able to actively take hand to contralateral axilla, corresponding to 115 of adduction and 55 degrees of forward flexion<br>Internal Rotation: Able to actively take hand to lower back OR ipsilateral back pocket, corresponding to 50 degrees of internal rotation<br>External Rotation: While standing with shoulder against a wall, able to actively take hand to the wall, corresponding to neutral external rotation.[2]								
**<u>SAKLAW NG PAGGALAW</u>**:<br>• Upang masuri ang iyong balikat, hihilingin naming gawin niyo ang ilang mga simpleng paggalaw.<br>• Bilugan ang Oo o Hindi sa mga susunod na katanungan. Ang lahat ng mga kilos na ito ay dapat gawin gamit ang apektadong balikat.<br>• Para sa unang bahagi, ikaw ay manatiling nakatayo o nakaupo nang nakasandal ang likod at ulo sa pader								
1. Kaya mo bang hawakan ang iyong dibdib?<br>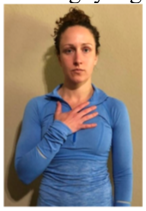<br>Oo Hindi								

2. Kaya mo bang hawakan ang iyong baba?<br>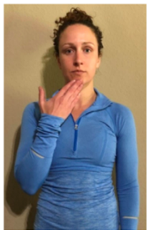<br>Oo Hindi								
3. Kaya mo bang hawakan ang iyong ilong?<br>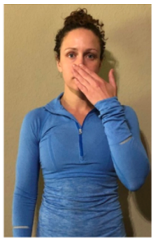<br>Oo Hindi								
4. Kaya mo bang hawakan ang tuktok ng iyong ulo?<br>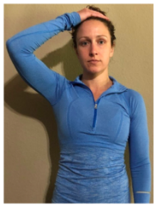<br>Oo Hindi								
5. Kaya mo bang hawakan ang likod ng iyong ulo?<br>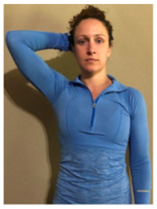<br>Oo Hindi								
6. Kaya mo bang abutin ang iyong kabilang kili-kili?<br>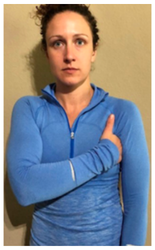<br>Oo Hindi								
7. Kaya mo bang abutin ang bulsa sa likod ng apektadong balikat?								

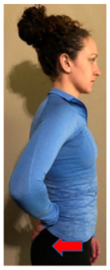<br>Oo Hindi								
8. Kaya mo bang abutin ang ibabang bahagi ng iyong likuran?<br>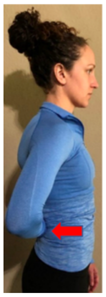<br>Oo Hindi								
9. Tumayo nang nakatagilid sa pader, siguradihin na ang apektadong balikat ang mas malapit sa pader. Itupi ang braso nang may 90 digris na anggulo sa iyong siko at subukang idikit ang kamay sa pader gamit ang likod ng iyong kamao. Kaya mo bang ilapat ang likod ng iyong kamay sa pader?<br>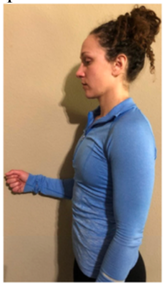 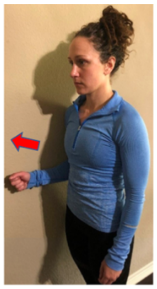<br>Oo Hindi								
**<u>Domain 4: Special Tests</u>**<br>Definition: “Musculoskeletal provocative maneuvers:”[1]<br>10. Drop arm for supraspinatus tear: Positive / Negative (Indicated by presence of absence of pain)<br>11. Cross Body for acromioclavicular joint dysfunction: Positive / Negative (self-performed without aid of additional examiner)<br>12. Belly Press for subscapularis dysfunction: Positive / Negative<br>13. Lift Off for subscapularis dysfunction: Positive / Negative<br>14. Speed’s for biceps tendon pathology: Positive / Negative (self-performed without aid of additional examiner) [2]								
ESPESYAL NA MGA PAGSUSURI:<br>• Para sa susunod na bahagi, hihilingin namin na gawin niyo ang ilang mga kilos na makakapagsabi sa amin kung aling mga galaw ang masakit para sa inyo. Ang mga kilos na ito ay gagamit ng magkabilang braso.								
10. Itaas ang inyong kamay sa gilid ng inyong katawan hanggang sa abot ng inyong makakaya. Sunod ay dahan-dahan itong ibaba. Masakit ba ang dahan-dahan na pagbaba ng braso?								

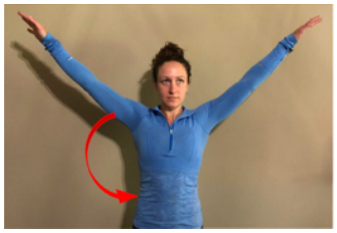 Oo Hindi								
11. Gamit ang masakit na balikat, umabot lagpas sa kabilang balikat at hilahin ito papalapit sa katawan gamit ang kamay sa normal na braso. Masakit ba ito? 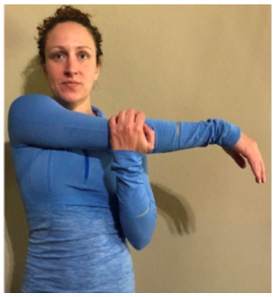 Oo Hindi								
12. Idiin ang kamay sa inyong tiyan. Masakit ba para sa apektadong balikat ang kilos na ito? 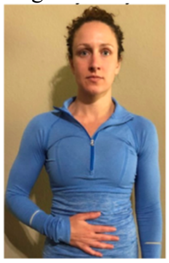 Oo Hindi								
13. Ilapat ang likod ng kamay sa ibabang bahagi ng inyong likod. Pagkatapos ay ilayo ito sa ibabang bahagi ng inyong likod. Masakit ba ito? 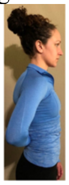 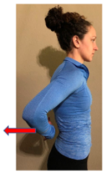 Oo Hindi								
14. Gamit ang masakit na balikat, itaas ang braso na parang may hawak na plato. Gamit ang kamay sa normal na braso, dahan-dahan itulak ang masakit na braso pababa at wag mo hayaan na ito ay bumaba. Masakit ba ito?								

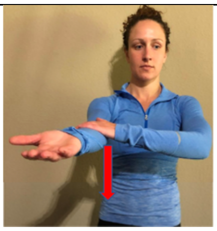<br>Oo Hindi								
**<u>Domain 5: Strength</u>**<br>Definition: “ability to voluntarily contract a muscle or muscle group at a specific joint”[1]<br>15. Internal rotators: Weak/ Painful/ Both/ Neither<br>16. External rotators: Weak/ Painful/ Both/ Neither<br>17. Abductors: Weak/ Painful/ Both/ Neither[2]								
**<u>Lakas</u>**<br>• Para sa susunod na bahagi, hihilingin naming na gawin niyo ang mga nakasaad na kilos sa apektadong balikat. Gamit ang kamay sa normal na braso, bigyan ng pwersa ang masakit na braso. Lahat ng ito ay dapat gawin na ang braso ay nakalapat lamang sa gilid ng katawan, ang siko ay nakatupi ng 90 digris na anggulo, at ang hinlalaki ay nakaturo sa taas.								
15. Gamit ang kamao sa normal na braso, idiin ito sa palad ng kamay sa masakit na braso. Mahina ba ang pakiramdaman ng iyong balikat? Masakit ba ito?<br>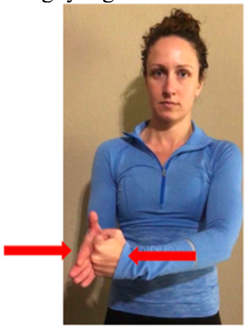<br>**Mahina Masakit Pareho Hindi**								
16. Gamit ang kamay sa normal na braso, hilahin ang apektadong kamay papunta sa direksyo ng normal na braso. Mahina ba ang pakiramdaman ng iyong balikat? Masakit ba ito?<br>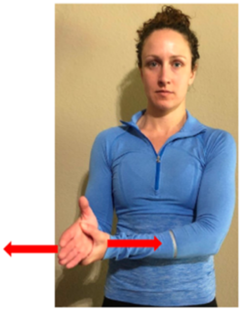<br>**Mahina Masakit Pareho Hindi**								
17. Gamit ang kamay sa normal na braso, kapitan ang siko sa apektadong braso. Gamit ang siko sa apektadong braso, ilayo ito mula sa katawan. Labanan ang kilos na ito gamit ang kamay sa normal na braso. Mahina ba ang pakiramdaman ng iyong balikat? Masakit ba ito?								

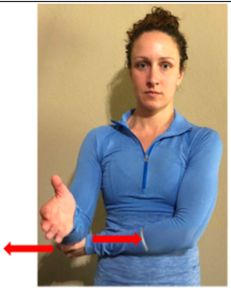 **Mahina Masakit Pareho Hindi**								

[1]Cifu, D et. al. (2021) Braddom's Physical Medicine and Rehabilitation. 6th edition. Elsevier Philadelphia, PA.

[2]Sprowls GR, Brown JC, Robin BN. The Shoulder Telehealth Assessment Tool in Transition to Distance Orthopedics. Arthroscopy Techniques. 2020 Nov 1;9(11):e1673– 81.
