# Supplementary material for: Linguistic Validation and Cross-Cultural Adaptation of the Shoulder Telehealth Assessment Tool for Filipino Patients with Musculoskeletal Shoulder Condition: Cross-Sectional Study
Source: JMIR Rehabil Assist Technol. 2026 Jan 20;13:e67974. doi: 10.2196/67974 (PMC12818489; doi:10.2196/67974)
Supplement: Multimedia Appendix 2 [file rehab-v13-e67974-s002.pdf]

Expert Consensus Committee Guide on Equivalence between English and Filipino versions

**Expert Consensus Committee Guide on Equivalence between English and Filipino versions:**

Please evaluate the reconciliated version for cultural equivalence through the following criteria. Please put a check on each box if these criteria are present.  You may put your rewording suggestions and other comments/ suggestions on the succeeding columns.

Semantic Equivalence (SE) – words used have similar, unique meaning in English and Filipino cultures

Idiomatic Equivalence (IE) – colloquialisms and idioms used are consistent or equivalent in English and Filipino cultures

Experiential Equivalence (EE) – experiences elicited are consistent or equivalent in English and Filipino cultures

Conceptual Equivalence (CE) – words or phrases used have consistent or equivalent conceptual meanings in English and Filipino cultures

| Original STAT | STAT – Filipino BT1 | STAT – Filipino BT2 | First Filipino Version of STAT | Cultural Equivalence | | | | Rewording Suggestion | Other Comments/ Suggestions |
|---|---|---|---|---|---|---|---|---|---|
| | | | | SE | IE | EE | CE | | |
| A. Instructions | | | | | | | | | |
| | | | | | | | | | |
| | | | | | | | | | |
| B. Item Content | | | | | | | | | |
| | | | | | | | | | |
| C. Recurring Choices | | | | | | | | | |
| | | | | | | | | | |
