# Supplementary material for: Linguistic Validation and Cross-Cultural Adaptation of the Shoulder Telehealth Assessment Tool for Filipino Patients with Musculoskeletal Shoulder Condition: Cross-Sectional Study
Source: JMIR Rehabil Assist Technol. 2026 Jan 20;13:e67974. doi: 10.2196/67974 (PMC12818489; doi:10.2196/67974)
Supplement: Multimedia Appendix 3 [file rehab-v13-e67974-s003.pdf]

Observation Checklist
Please put a check mark on the corresponding performance of each task accordingly.

A. Without Pictorial Guide

Participant code no:	Observer:			Date:	
Observation Checklist *Actual data in Filipino STAT version*	Participant Response	Performs the task correctly without pictorial guide	Needs cuing from caregiver to perform the task correctly	Does not perform the task correctly	Remarks (qualitative description of shoulder movement/ compensations/ unable to perform task due to severe pain, etc.)

B. With Pictorial Guide

Participant code no:	Observer:			Date:	
Observation Checklist *Actual data in Filipino STAT version*	Participant Response	Performs the task correctly with pictorial guide	Needs cuing from caregiver to perform the task correctly	Does not perform the task correctly	Remarks (qualitative description of shoulder movement/ compensations/ unable to perform task due to severe pain, etc.)
