# Supplementary material for: Linguistic Validation and Cross-Cultural Adaptation of the Shoulder Telehealth Assessment Tool for Filipino Patients with Musculoskeletal Shoulder Condition: Cross-Sectional Study
Source: JMIR Rehabil Assist Technol. 2026 Jan 20;13:e67974. doi: 10.2196/67974 (PMC12818489; doi:10.2196/67974)
Supplement: Multimedia Appendix 4 [file rehab-v13-e67974-s004.pdf]

Open-Ended Interview Form

Participant code number:	Interviewer:	Date:
Questions:	Response	
1. Paano mo mailalarawan ang iyong karanasan sa paggamit ng STAT?		
2. Anu-anong mga bagay ang nakatulong sa iyo sa paggamit ng STAT?		
3. Anu-anong mga bagay ang nakahadlang sa iyo sa paggamit ng STAT?		
4. Anu-ano ang iyong mga suhestyon para mapaigi ang paggamit ng STAT?		
