# Supplementary material for: Linguistic Validation and Cross-Cultural Adaptation of the Shoulder Telehealth Assessment Tool for Filipino Patients with Musculoskeletal Shoulder Condition: Cross-Sectional Study
Source: JMIR Rehabil Assist Technol. 2026 Jan 20;13:e67974. doi: 10.2196/67974 (PMC12818489; doi:10.2196/67974)
Supplement: Multimedia Appendix 5 [file rehab-v13-e67974-s005.pdf]

The Shoulder Telehealth Assessment Tool (STAT)

<u>PANANAKIT AT MGA GAWAIN</u>

1. Gaano katindi ang sakit ng inyong balikat? Pakibilugan ang numerong katumbas ng karaniwang lebel ng sakit na nararamdaman sa araw-araw:

0	1	2	3	4	5	6	7	8	9	10
**Walang Sakit**										**Pinaka-masakit**

2. Kung 100% ang katumbas ng normal, mula 0% hanggang 100%, gaano ka-normal ang pakiramdam ng inyong apektadong balikat ngayon? Pakibilugan ang naaangkop na porsyento.

0	10	20	30	40	50	60	70	80	90	100
**Hindi Normal**										**Normal**

3. Gaano naaapektuhan ng sakit ng balikat ang inyong pang-araw-araw naaktibidad?

   a. Pakibilugan ang inyong kasalukuyang lebel ng **<u>pagtulog</u>**:

0	1	2	3	4	5	6	7	8	9	10
**Hindi Naaapektuhan**										**Lubhang Apektado**

   b. Pakibilugan ang inyong kasalukuyang lebel ng **<u>paggawa ng gawaing bahay o trabaho</u>**:

0	1	2	3	4	5	6	7	8	9	10
**Hindi Naaapektuhan**										**Lubhang Apektado**

   c. Pakibilugan ang inyong kasalukuyang lebel ng **<u>panglibangan na aktibidad o isports</u>**:

0	1	2	3	4	5	6	7	8	9	10
**Hindi Naaapektuhan**										**Lubhang Apektado**

Kung hindi naaangkop, maaaring tumunga sa susunod na bahagi

SAKLAW NG PAGGALAW:

- Upang masuri ang inyong balikat, hihilingin naming gawin ninyo ang ilang mga simpleng paggalaw.
- Bilugan ang Oo o Hindi sa mga susunod na katanungan. Ang lahat ng mga kilos na ito ay dapat gawin gamit ang apektadong balikat.
- Para sa unang bahagi, ikaw ay manatiling nakatayo o nakaupo nang nakasandal ang likod at ulo sa pader.

1. Kaya mo bang hawakan ang inyong dibdib?

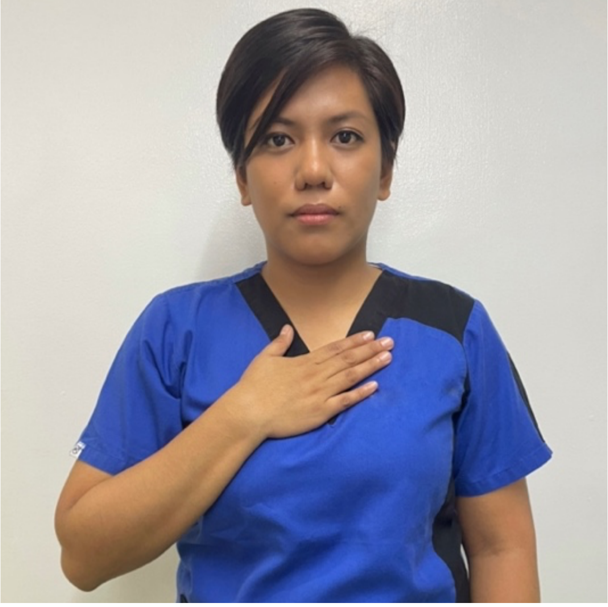

Oo **Hindi**

2. Kaya mo bang hawakan ang inyong baba?

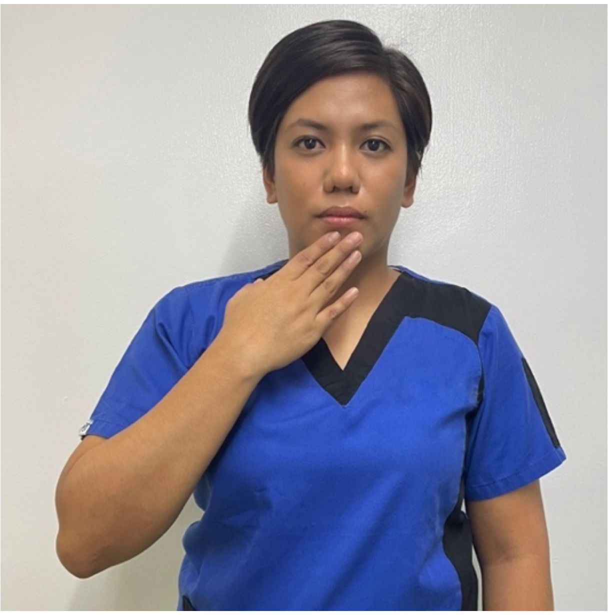

Oo **Hindi**

3. Kaya mo bang hawakan ang inyong ilong?

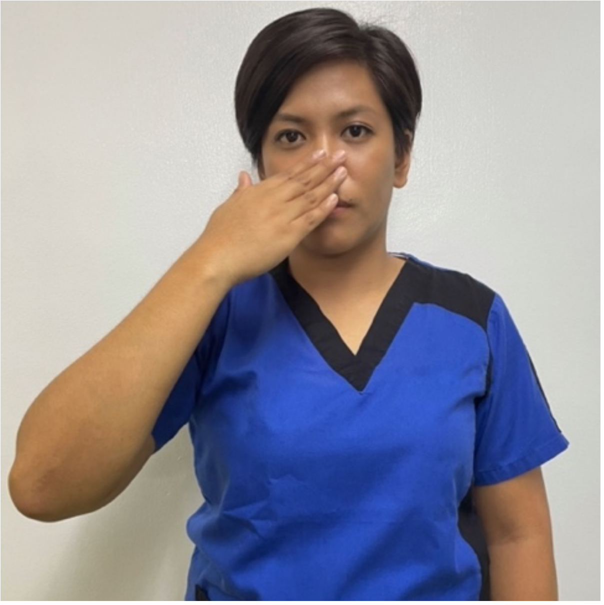

Oo **Hindi**

4. Kaya mo bang hawakan ang tuktok ng inyong ulo?

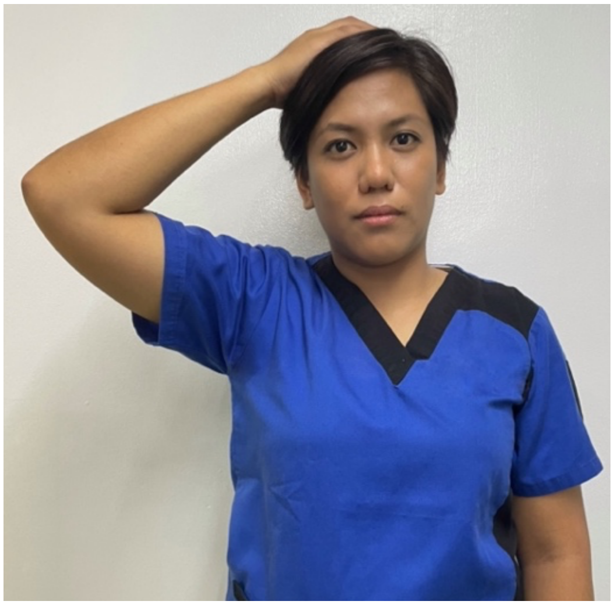

Oo **Hindi**

5. Kaya mo bang hawakan ang likod ng inyong ulo?

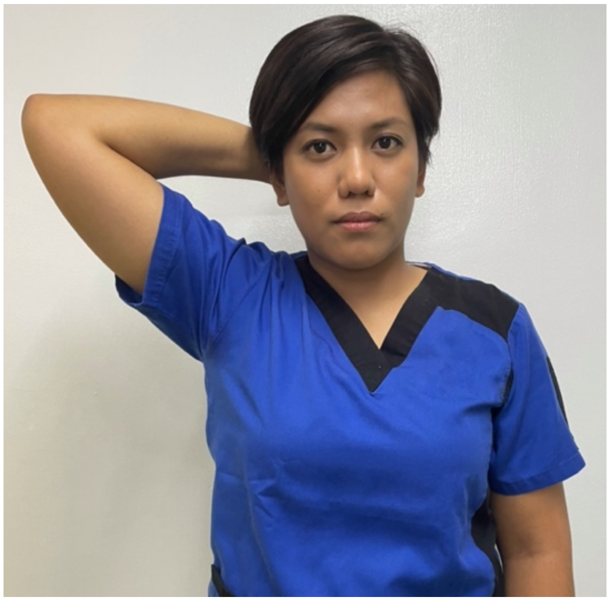

Oo **Hindi**

6. Kaya mo bang abutin ang inyong kabilang kili-kili?

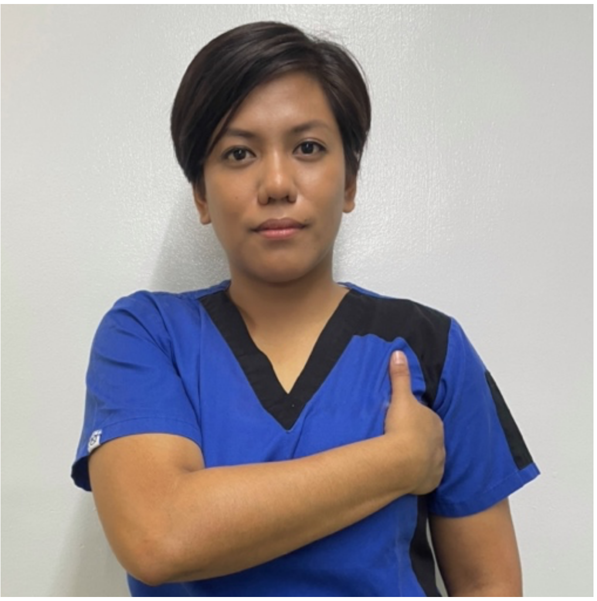

Oo **Hindi**

7. Gamit ang likod ng kamay, kaya mo bang abutin ang inyong pigi sa likod ng apektadong balikat?

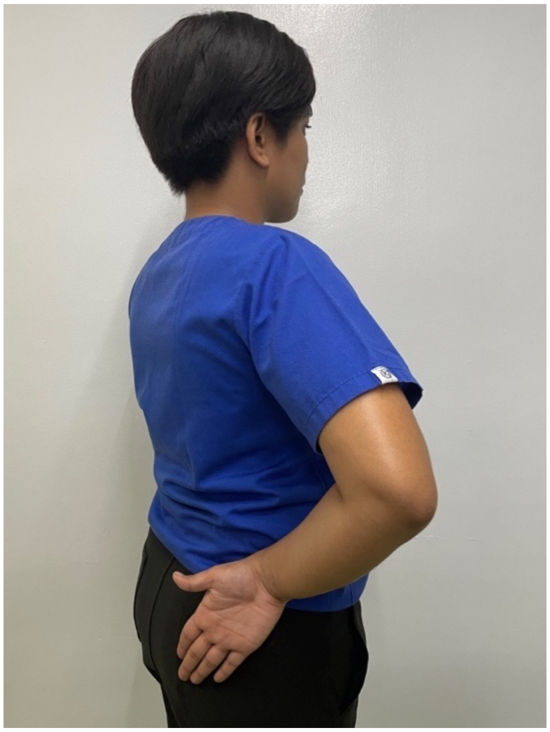

Oo **Hindi**

8. Gamit ang likod ng kamay, kaya mo bang abutin ang ibabang bahagi ng inyong likuran?

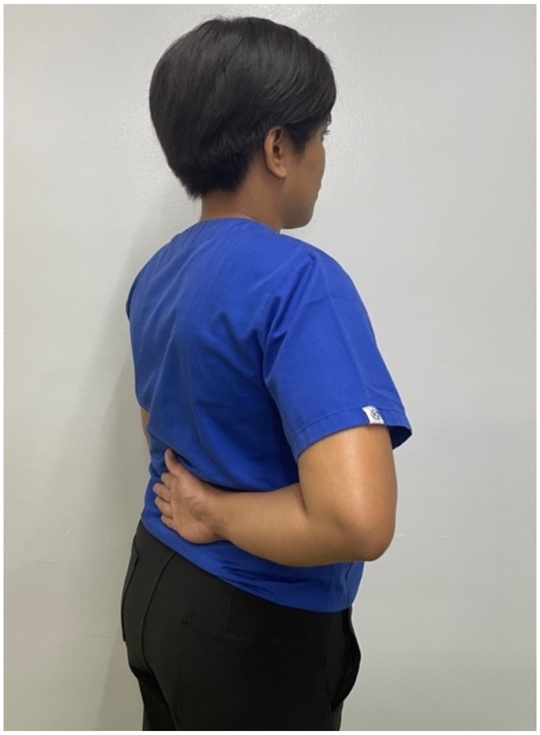

Oo **Hindi**

9. A. Tumayo nang nakatagilid sa pader at siguraduhin na ang apektadong balikat ang mas malapit sa pader.

B. Itupi ang siko nang may 90 digris na anggulo

C. Subukang idikit ang kamay sa pader gamit ang likod ng inyong kamao.

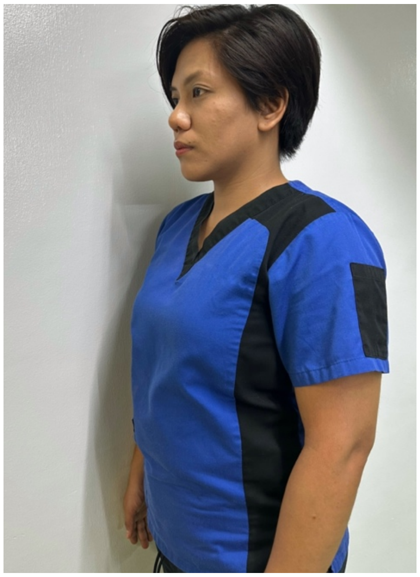

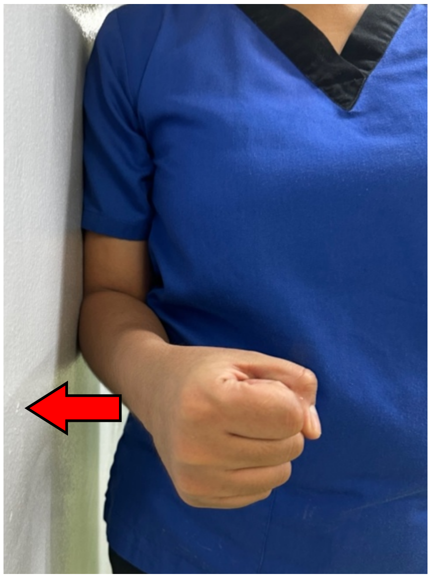

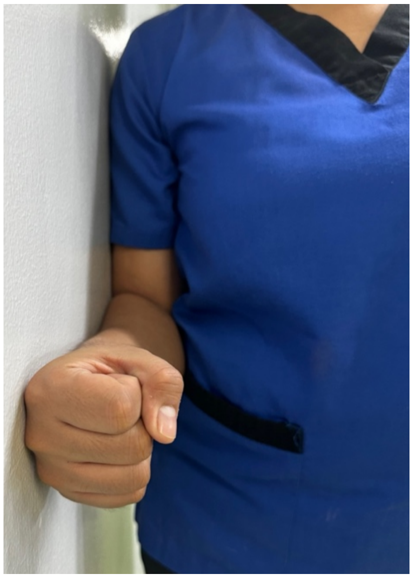

Kaya mo bang ilapat ang likod ng inyong kamay sa pader?

Oo **Hindi**

LAKAS

- Para sa susunod na bahagi, hihilingin namin na gawin niyo ang mga nakasaad na kilos sa apektadong balikat. Gamit ang kamay sa normal na braso, labanan ang masakit na braso.

10. A. Ilapat ang braso sa gilid ng katawan

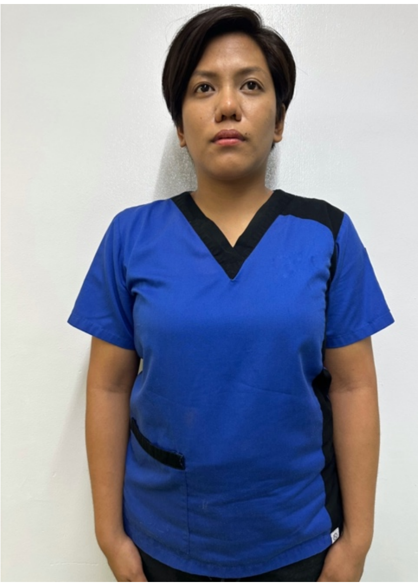

B. Itupi ang siko nang may 90 digris na anggulo at ang hinlalaki ay nakaturo sa taas

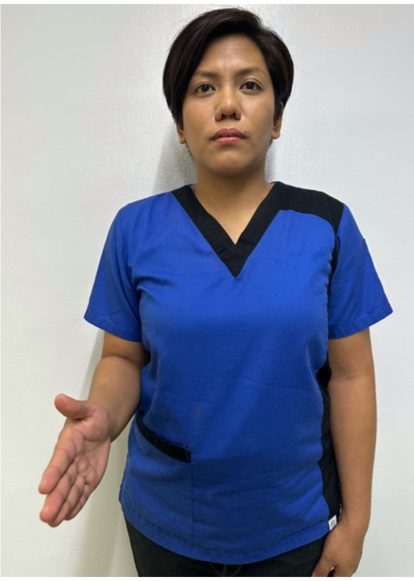

C. Gamit ang palad ng apektadong braso, ilaban ito sa kamao ng normal na braso papasok sa tyan.

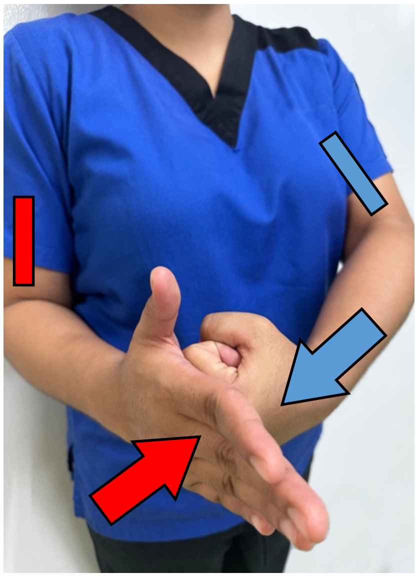

Normal na braso
Apektadong braso

Pakiramdam mo ba mahina ang apektadong balikat? Masakit ba ito?

Mahina **Masakit** **Pareho** **Hindi**

11. A. Ilapat ang braso sa gilid ng katawan

B. Itupi ang siko nang may 90 digris na anggulo at ang hinlalaki ay nakaturo sa taas

C. Gamit ang likod ng kamay ng apektadong braso, ilaban ito sa kamay ng normal na braso papalayo sa tyan.

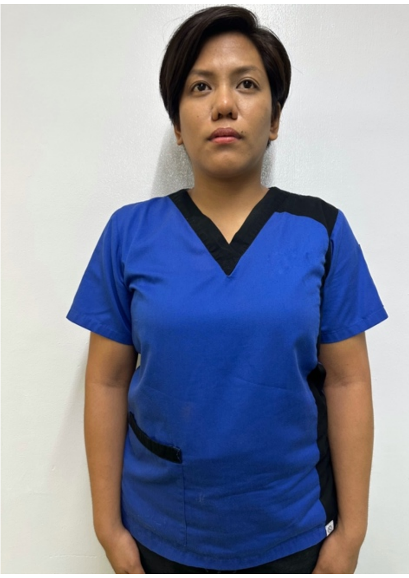

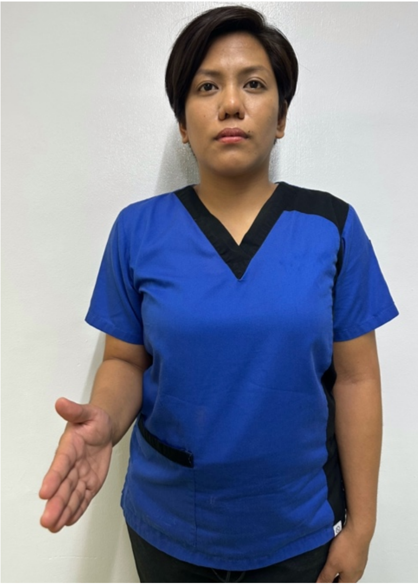

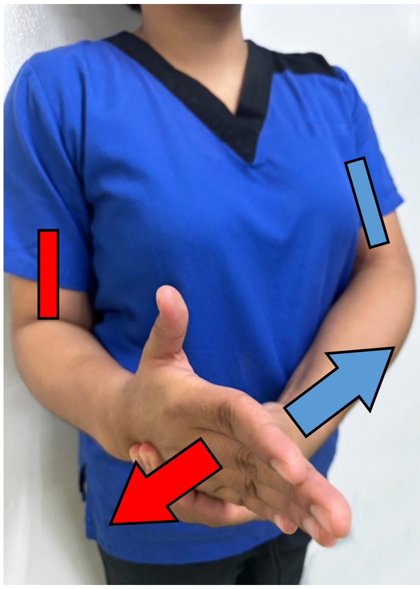

Normal na braso

Apektadong braso

Pakiramdam mo ba mahina ang apektadong balikat? Masakit ba ito?

Mahina **Masakit** **Pareho** **Hindi**

12. A. Ilapat ang braso sa gilid ng katawan

B. Itupi ang siko nang may 90 digris na anggulo at ang hinlalaki ay nakaturo sa taas

C. Ikawit nang mahigpit ang siko sa apektadong braso. Gamit ang siko sa apektadong braso, ilayo ito mula sa katawan habang pinipigilan ng kamay ng normal na braso. .

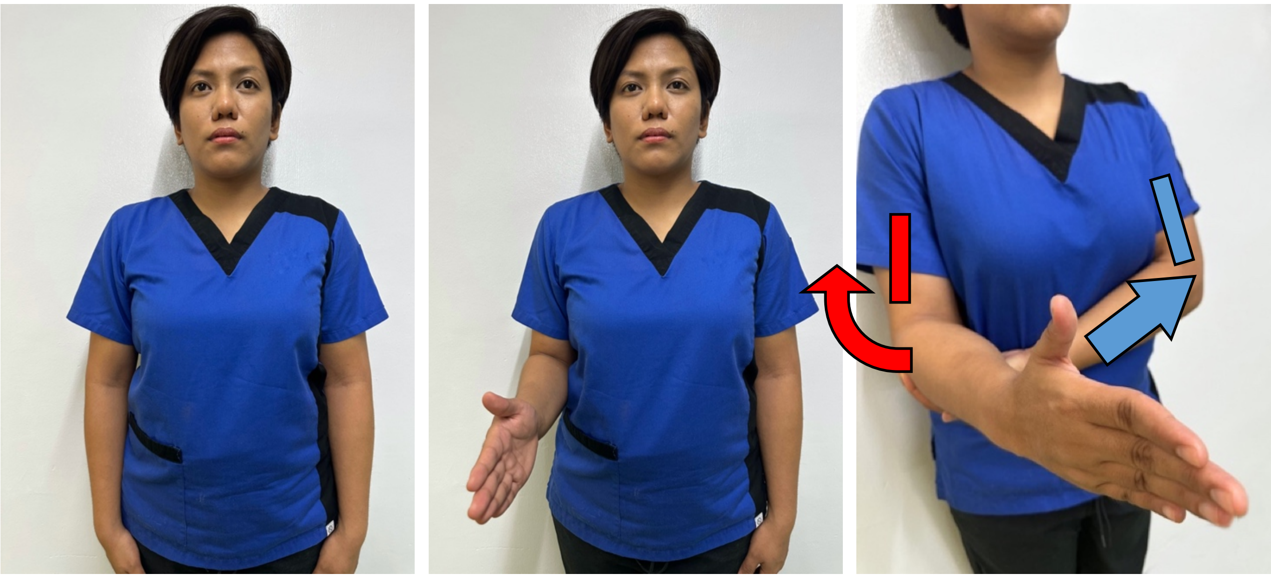

Normal na braso

Apektadong braso

Pakiramdam mo ba mahina ang apektadong balikat? Masakit ba ito?

Mahina **Masakit** **Pareho** **Hindi**

ESPESYAL NA MGA PAGSUSURI

- Para sa susunod na bahagi, hihilingin namin na gawin ninyo ang ilang mga kilos upang malaman namin kung aling mga galaw ang masakit para sa apektadong balikat. Ang mga kilos na ito ay gagamit ng magkabilang braso.
- Maaaring makaramdam ng sakit habang ginagawa ang mga kilos. Mangyari lamang na sabihin ninyo kaagad sa amin at itigil ninyo ang kilos kung may masakit.

13. A. Itaas ang inyong kamay sa gilid ng inyong katawan hanggang sa abot ng inyong makakaya.

B. Pagkatapos ay dahan-dahan itong baba.

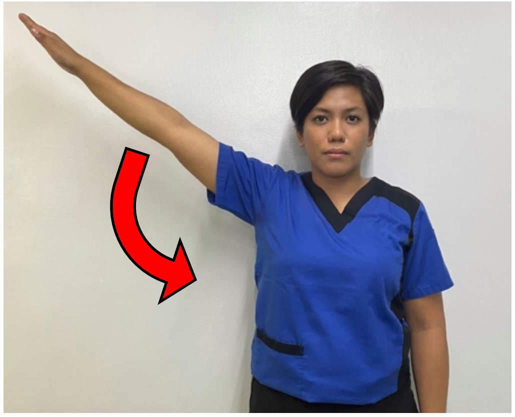

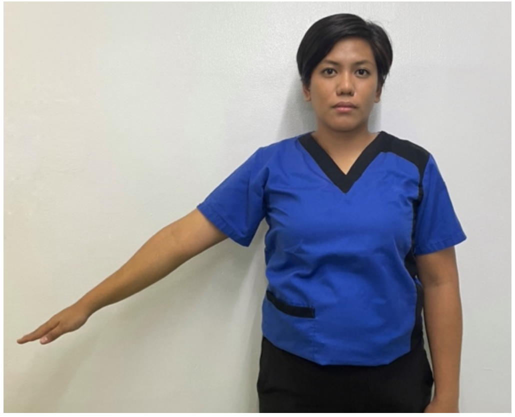

Kung masakit ang balikat, maaaring alalayan ng apektadong balikat sa kabilang kamay.

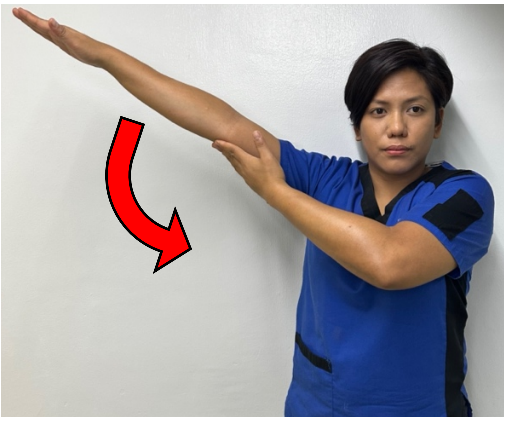

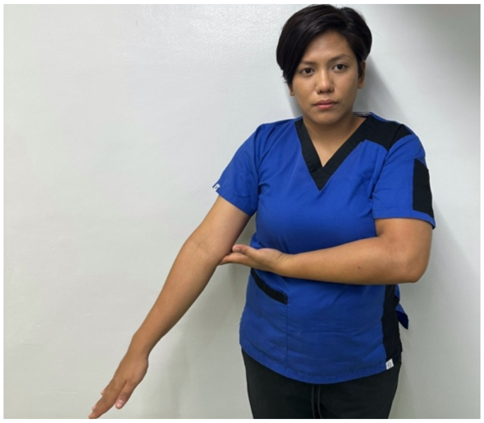

Masakit ba ang balikat sa dahan-dahan na pagbaba ng braso?

Oo **Hindi**

14. Gamit ang masakit na balikat, abutin ang kabilang bahagi ng katawan nang nakaunat ang siko. Hilahin ito papalapit sa katawan gamit ang kamay sa normal na braso. Masakit ba ang apektadong balikat sa posisyong ito?

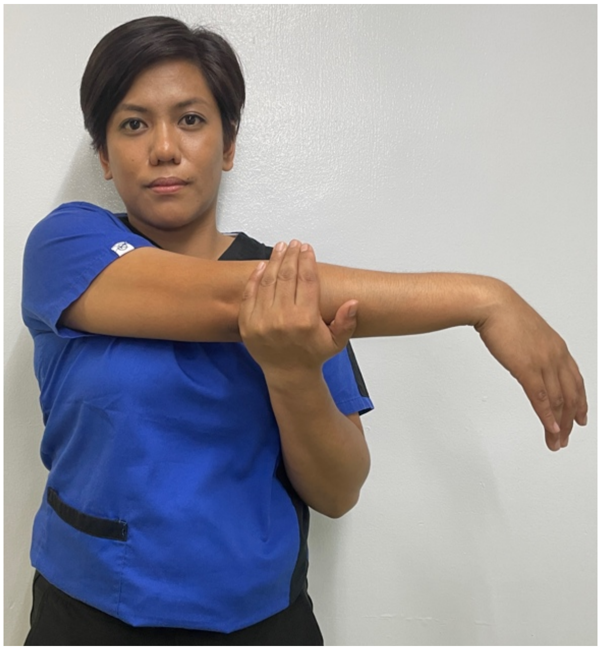

Oo **Hindi**

15. Idiin ang kamay ng masakit na braso sa inyong tiyan. Masakit ba ang apektadong balikat sa posisyong ito?

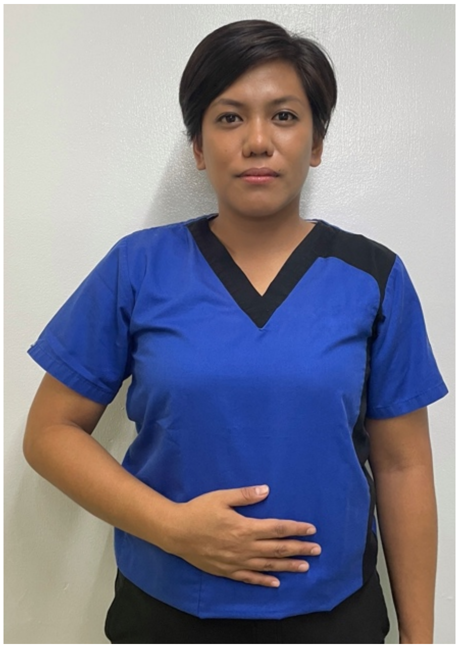

Oo **Hindi**

16. A. Ilapat ang likod ng kamay sa ibabang bahagi ng inyong likod.

B. Pagkatapos ay ilayo ito.

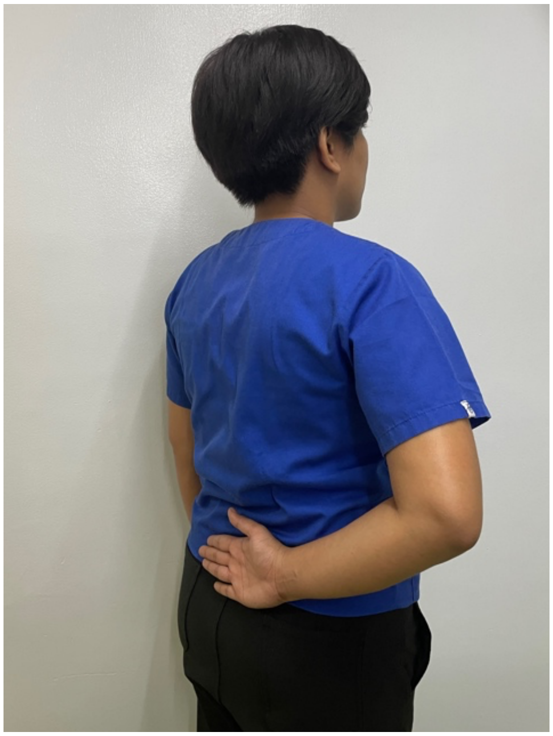

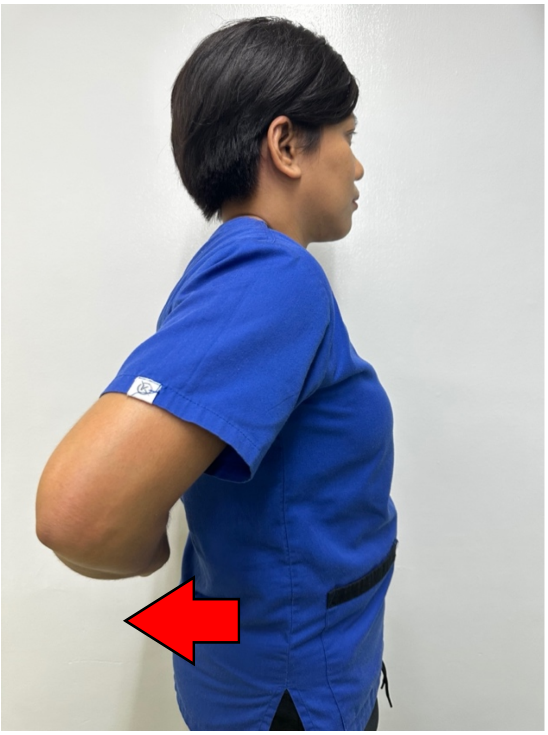

Masakit ba ang apektadong balikat sa posisyong ito?

Oo **Hindi**

17. A. Gamit ang masakit na balikat, itaas ang braso paharap sa lebel ng inyong balikat na parang nanghihingi habang nakaunat ang inyong siko.

B. Gamit ang kamay sa normal na braso, dahan-dahan itulak ang masakit na braso pababa at wag mo hayaan na ito ay bumaba.

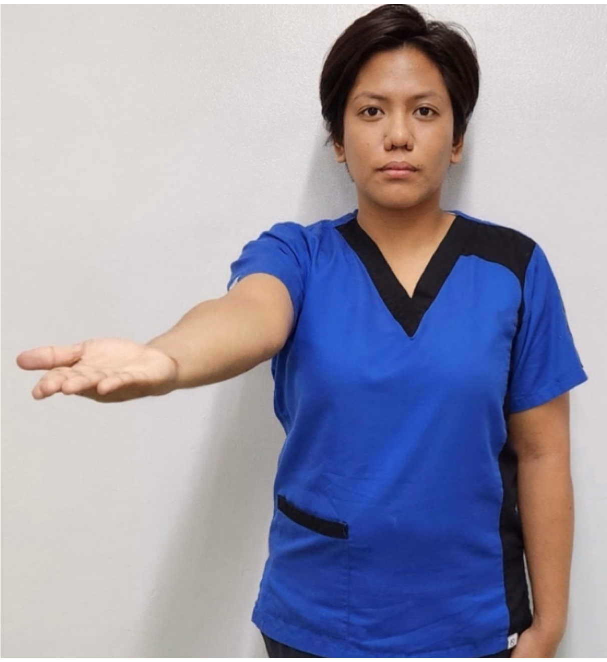

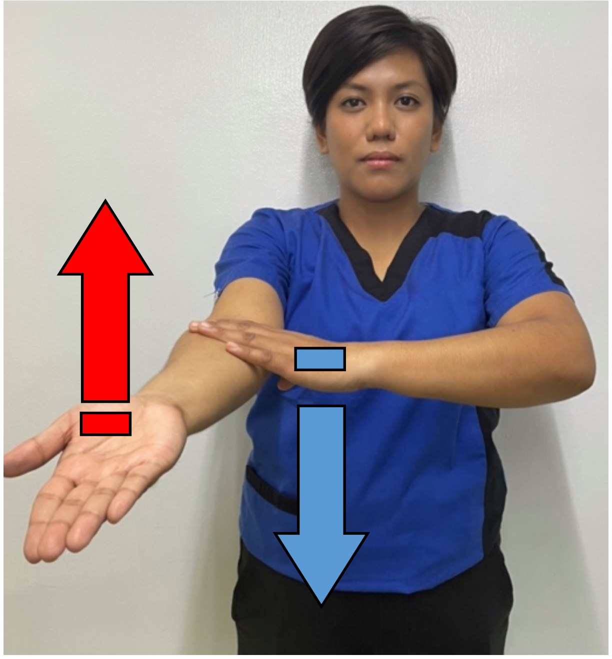

Normal na balikat

Apektadong balikat

Masakit ba ang apektadong balikat sa posisyong ito?

Oo **Hindi**
