# Supplementary material for: Linguistic Validation and Cross-Cultural Adaptation of the Shoulder Telehealth Assessment Tool for Filipino Patients with Musculoskeletal Shoulder Condition: Cross-Sectional Study
Source: JMIR Rehabil Assist Technol. 2026 Jan 20;13:e67974. doi: 10.2196/67974 (PMC12818489; doi:10.2196/67974)
Supplement: Multimedia Appendix 6 [file rehab-v13-e67974-s006.docx]

**Supplemental** **table 1. Content Validity Index**

| **Item** | **Expert 1** | **Expert 2** | **Expert 3** | **Expert 4** | **Expert 5** | **Expert 6** | **Experts in Agreement** | **I-CVI** | **UA** | **Kappa** |
| --- | --- | --- | --- | --- | --- | --- | --- | --- | --- | --- |
| **Pain and Activity** | | | | | | | | | | |
| Q1 | 1 | 1 | 1 | 1 | 1 | 1 | 6 | 1.00 | 1 | 1.00 |
| Q2 | 1 | 1 | 1 | 1 | 1 | 1 | 6 | 1.00 | 1 | 1.00 |
| Q3 | 1 | 1 | 1 | 1 | 1 | 1 | 6 | 1.00 | 1 | 1.00 |
| **Range of Motion** | | | | | | | | | | |
| Q1 | 0 | 1 | 1 | 1 | 1 | 1 | 5 | 0.83 | 0 | 0.82 |
| Q2 | 0 | 1 | 1 | 1 | 1 | 1 | 5 | 0.83 | 0 | 0.82 |
| Q3 | 0 | 1 | 1 | 1 | 1 | 1 | 5 | 0.83 | 0 | 0.82 |
| Q4 | 1 | 1 | 1 | 1 | 1 | 1 | 6 | 1.00 | 1 | 1.00 |
| Q5 | 1 | 1 | 1 | 1 | 1 | 1 | 6 | 1.00 | 1 | 1.00 |
| Q6 | 1 | 1 | 1 | 1 | 1 | 1 | 6 | 1.00 | 1 | 1.00 |
| Q7 | 1 | 1 | 1 | 1 | 1 | 1 | 6 | 1.00 | 1 | 1.00 |
| Q8 | 1 | 1 | 1 | 1 | 1 | 1 | 6 | 1.00 | 1 | 1.00 |
| Q9 | 1 | 1 | 0 | 1 | 1 | 1 | 5 | 0.83 | 0 | 0.82 |
| **Strength** | | | | | | | | | | |
| Q10 | 1 | 1 | 1 | 1 | 1 | 1 | 6 | 1.00 | 1 | 1.00 |
| Q11 | 1 | 1 | 1 | 1 | 1 | 1 | 6 | 1.00 | 1 | 1.00 |
| Q12 | 1 | 1 | 1 | 1 | 1 | 1 | 6 | 1.00 | 1 | 1.00 |
| **Special Tests** | | | | | | | | | | |
| Q13 | 1 | 1 | 1 | 1 | 1 | 1 | 6 | 1.00 | 1 | 1.00 |
| Q14 | 1 | 1 | 1 | 1 | 1 | 1 | 6 | 1.00 | 1 | 1.00 |
| Q15 | 1 | 1 | 1 | 1 | 1 | 1 | 6 | 1.00 | 1 | 1.00 |
| Q16 | 1 | 1 | 1 | 1 | 1 | 1 | 6 | 1.00 | 1 | 1.00 |
| Q17 | 1 | 1 | 1 | 1 | 1 | 1 | 6 | 1.00 | 1 | 1.00 |
|  |  |  |  |  |  |  | **S-CVI/Ave** | 0.97 |  |  |
|  |  |  |  |  |  |  | **S-CVI/UA** |  | 0.80 |  |

*I-CVI = item-level content validity index; UA = universal agreement; S-SCI = scale-level content validity index; Ave = average*

**Supplemental table 2. Pre-Test Observation Checklist**

| **Item** | **Without Picture** | | | **With Picture** | | | **Remarks/Mistakes** |
| --- | --- | --- | --- | --- | --- | --- | --- |
|  | **P**  **n (%)** | **C**  **n (%)** | **D**  **n (%)** | **P**  **n (%)** | **C**  **n (%)** | **D**  **n (%)** |  |
| **Pain & Activity** | | | | | | | |
| Q1 | 11(91.7) | 1(8.3) | 0(0) | - | - | - |  |
| Q2 | 8(66.7) | 3(0.25) | 1(8.3) | - | - | - | Mistaken as a reversed scale |
| Q3A | 10(83.3) | 2(16.7) | 0(0) | - | - | - | Skips this question |
| Q3B | 10(83.3) | 2(16.7) | 0(0) | - | - | - |  |
| Q3C | 10(83.3) | 2(16.7) | 0(0) | - | - | - | Answers despite not engaged in activities |
| **Range of Motion** | | | | | | | |
| Q1 | 8(66.7) | 0(0) | 4(33.3) | 11(91.7) | 0(0) | 1(8.3) | Touches heart instead of middle chest |
| Q2 | 12(100) | 0(0) | 0(0) | 12(100) | 0(0) | 0(0) |  |
| Q3 | 12(100) | 0(0) | 0(0) | 12(100) | 0(0) | 0(0) |  |
| Q4 | 11(91.7) | 0(0) | 1(8.3) | 12(100) | 0(0) | 0(0) | Touches nape instead top of head |
| Q5 | 11(91.7) | 0(0) | 1(8.3) | 12(100) | 0(0) | 0(0) | Touches occiput instead of nape |
| Q6 | 12(100) | 0(0) | 0(0) | 12(100) | 0(0) | 0(0) |  |
| Q7 | 7(58.3) | 0(0) | 5(41.7) | 11(91.7) | 0(0) | 1(8.3) | Puts palm on buttocks |
| Q8 | 2(16.7) | 0(0) | 10(83.3) | 11(91.7) | 0(0) | 1(8.3) | Puts palm on back/ reaches upper back |
| Q9 | 1(8.3) | 0(0) | 11(91.7) | 8(66.7) | 0(0) | 4(33.3) | Inconsistent responses despite picture |
| **Strength** | | | | | | | |
| Q10 | 2(16.7) | 0(0) | 10(83.3) | 7(58.3) | 0(0) | 5(41.7) | Inconsistent responses despite picture |
| Q11 | 1(8.3) | 0(0) | 11(91.7) | 3(25) | 0(0) | 0(0) | Inconsistent responses despite picture |
| Q12 | 2(16.7) | 0(0) | 10(83.3) | 7(58.3) | 0(0) | 5(41.7) | Inconsistent responses despite picture |
| **Special Test** | | | | | | | |
| Q13 | 3(25) | 0(0) | 9(0.75) | 11(91.7) | 0(0) | 1(8.3) | Shoulder flexed forward  Does not descend slowly  Confused with conditional statement |
| Q14 | 0(0) | 1(8.3) | 11(91.7) | 9(75) | 0(0) | 3(25) | Reaches contralateral shoulder |
| Q15 | 11(91.7) | 0(0) | 1(8.3) | 12(100) | 0(0) | 0(0) |  |
| Q16 | 5(41.7) | 0(0) | 7(58.3) | 6(50) | 0(0) | 6(50) | Does not lift off despite picture |
| Q17 | 0(0) | 0(0) | 12(100) | 11(91.7) | 0(0) | 1(8.3) | Position arm abducted, elbow flexed |

**Supplemental table 3. Pilot Study Observational Checklist**

| **Item** | **Without Picture** | | | **With Picture** | | |
| --- | --- | --- | --- | --- | --- | --- |
|  | **Performs the task correctly without pictorial guide n (%)** | **Needs cuing from caregiver to perform task correctly n (%)** | **Does not perform task correctly n (%)** | **Performs the task correctly with pictorial guide n (%)** | **Needs cuing from caregiver to perform task correctly n (%)** | **Does not perform task correctly n (%)** |
| **Pain and Activity** |  |  |  |  |  | - |
| Q1 | 43 (91.5) | 3 (6.4) | 0 (0) | - | - | - |
| Q2 | 35 (74.5) | 11 (23.4) | 1 (2.1) | - | - | - |
| Q3A | 42 (89.4) | 5 (10.6) | 0 (0) | - | - | - |
| Q3B | 42 (89.4) | 5 (10.6) | 0 (0) | - | - | - |
| Q3C | 40 (85.1) | 6 (12.8) | 1 (2.1) | - | - | - |
| **Range of Motion** |  |  |  |  |  |  |
| Q1 | 43 (91.5) | 1 (2.1) | 3 (6.4) | 46 (97.9) | 0 (0) | 1 (2.1) |
| Q2 | 36 (76.6) | 0 (0) | 11 (23.4) | 45 (95.7) | 0 (0) | 2 (4.3) |
| Q3 | 47 (100) | 0 (0) | 0 (0) | 47 (100) | 0 (0) | 0 (0) |
| Q4 | 41 (87.2) | 1 (2.1) | 5 (10.6) | 46 (97.9) | 0 (0) | 1 (2.1) |
| Q5 | 44 (93.6) | 1 (2.1) | 2 (4.3) | 45 (95.7) | 0 (0) | 2 (4.3) |
| Q6 | 45 (95.7) | 0 (0) | 2 (4.3) | 46 (97.9) | 0 (0) | 1 (2.1) |
| Q7 | 14 (29.8) | 1 (2.1) | 32 (68.1) | 35 (74.5) | 4 (8.5) | 8 (17) |
| Q8 | 21 (44.7) | 4 (8.5) | 22 (46.8) | 38 (80.9) | 2 (4.3) | 7 (14.9) |
| Q9 | 5 (10.6) | 0 (0) | 42 (89.4) | 35 (74.5) | 6 (12.8) | 6 (12.8) |
| **Strength** |  |  |  |  |  |  |
| Q10 | 10 (21.3) | 3 (6.4) | 34 (72.3) | 27 (57.4) | 8 (17) | 12 (25.5) |
| Q11 | 9 (19.1) | 0 (0) | 38 (80.9) | 15 (31.9) | 2 (4.3) | 30 (63.8) |
| Q12 | 12 (25.5) | 2 (4.3) | 32 (68.1) | 12 (25.5) | 3 (6.4) | 31 (66) |
| **Special Tests** |  |  |  |  |  |  |
| Q13 | 14 (29.8) | 2 (4.3) | 30 (63.8) | 41 (87.2) | 4 (8.5) | 1 (2.1) |
| Q14 | 11 (23.4) | 0 (0) | 35 (74.5) | 35 (74.5) | 6 (12.8) | 5 (10.6) |
| Q15 | 33 (70.2) | 2 (4.3) | 11 (23.4) | 41 (87.2) | 5 (10.6) | 0 (0) |
| Q16 | 26 (55.3) | 2 (4.3) | 18 (38.3) | 39 (83) | 2 (4.3) | 5 (10.6) |
| Q17 | 15 (31.9) | 4 (8.5) | 27 (57.4) | 34 (72.3) | 5 (10.6) | 7 (14.9) |

**Supplemental table 4. Measure of Internal Consistency**

|  | **Cronbach's Alpha value** |
| --- | --- |
| **Overall** | **0.8692** |
| **Pain and Activity** |  |
| Q1 | 0.8628 |
| Q2 | 0.8717 |
| Q3A | 0.8649 |
| Q3B | 0.8607 |
| Q3C | 0.8634 |
| **Range of Motion** |  |
| Q4 | 0.863 |
| Q5 | 0.863 |
| Q7 | 0.8703 |
| Q8 | 0.8589 |
| Q9 | 0.863 |
| **Strength** |  |
| Q10 | 0.8552 |
| Q11 | 0.8597 |
| Q12 | 0.8595 |
| **Special Tests** |  |
| Q13 | 0.8587 |
| Q14 | 0.863 |
| Q15 | 0.8641 |
| Q16 | 0.8594 |
| Q17 | 0.8626 |
